# Timely Recognition of Abusive Injuries (TRAIN): Results from a Statewide Quality Improvement Collaborative

**DOI:** 10.1097/pq9.0000000000000637

**Published:** 2023-04-10

**Authors:** Kristin Garton Crichton, Sandra Spencer, Robert Shapiro, Paul McPherson, Eugene Izsak, Lolita M. McDavid, Carrie Baker, Jonathan D. Thackeray

**Affiliations:** From the * Department of Pediatrics, Division of Child and Family Advocacy, Nationwide Children’s Hospital, Columbus, Ohio; † Department of Pediatrics, Section of Emergency Medicine, Children’s Hospital Colorado, Denver, Colorado; ‡ Department of Clinical Pediatrics, University of Cincinnati College of Medicine, Cincinnati Children’s Hospital Medical Center, Cincinnati, Ohio; § Department of Pediatrics, Division of Child Protection and Child Abuse Prevention, Akron Children’s Hospital, CARE Center, Akron, Ohio; ¶ Pediatric Emergency Medicine, ProMedica Russell J. Ebeid Children’s Hospital, Toledo, Ohio; ‖ Department of Pediatrics, Rainbow Babies and Children’s Hospital, Cleveland, Ohio; ** Health Impact Ohio, Columbus, Ohio; †† Department of Pediatrics, Division of Child and Family Advocacy, Dayton Children’s Hospital, Dayton, Ohio.

## Abstract

Early recognition of physical abuse is critical as children often experience recurrent abuse if their environment remains unchanged. The Timely Recognition of Abusive Injuries (TRAIN) Collaborative was a quality improvement network of 6 Ohio children’s hospitals created in 2015 to improve the management of injuries concerning for abuse in infants. TRAIN’s first phase sought to reduce recurrent abuse by recognizing and responding to injured infants. This study aimed to reduce reinjury rate among infants ≤6 months by 10% at 1 year and 50% by 2 years and sustain improvement for 1 year as reflected in 3- and 12-month reinjury rates.

**Methods:**

The TRAIN Collaborative adopted the Institute for Healthcare Improvement’s Breakthrough Series Collaborative Model, where partnerships between organizations facilitate learning from each other and experts. Collaborative members identified opportunities to improve injury recognition, implemented changes, responded to data, and reconvened to share successes and obstacles. As a result, institutions implemented different interventions, including education for clinical staff, increased social work involvement, and scripting for providers.

**Results:**

Data collected over 3 years were compared to a 12-month baseline. The number of injuries increased from 51 children with concerning injuries identified monthly to 76 children sustained throughout the collaborative. However, within 2 years, the 3- and 12-month reinjury rates ultimately significantly decreased from 5.7% to 2.1% and 6.5% to 3.7%, respectively.

**Conclusion:**

Our data suggest the Institute for Healthcare Improvement’s Breakthrough Series model can be applied across large populations to improve secondary injury prevention in infants.

## INTRODUCTION

In 2020, >600,000 children were victims of physical abuse, including 1750 fatalities.^1^ Early recognition of signs of physical abuse is critical to prevent continuing or escalating abuse, as recurrent abusive events are common if the child’s environment remains unchanged.^2,3^ Recurrent physical abuse occurs in 30%–50% of cases, and abusive injuries often increase in severity over time.^2,3^ The term “sentinel injury” (SI) was first published in 2013 to describe those abusive injuries, often relatively minor in severity, that may precede more severe recurrent abuse. Sheets et al^4^ (2013) retrospectively reviewed 401 infants ≤12 months for the presence of SI, which they defined as “a previous injury reported in the medical history that was concerning for abuse because the infant could not cruise, or the explanation was implausible.” Two hundred of these infants were determined to be “definitely abused” in this retrospective study, 63 of whom had a previous SI noted by a caregiver or health care provider, as compared to no SI noted in 101 “nonabused” controls. A subsequently published retrospective review of pediatric Medicaid claims in Ohio data found that the presence of any minor injury, even as slight as a small laceration, was an independent risk factor for an event of concern for recurrent abuse.^2^

Unfortunately, significant variability exists in providers’ ability to recognize abusive injury.^5–9^ Furthermore, while the American Academy of Pediatrics has established guidelines for evaluating suspected abusive injuries, significant variation exists in the consistency of medical evaluation and reporting of concerning injuries once identified.^10–14^

Morbidity and mortality increase with recurrent episodes of physical abuse. One study demonstrated a mortality rate as high as 24.5% in children who suffered from recurrent abuse compared with 9.9% in children with a single episode of abuse.^12^ Ohio child abuse pediatricians engaged in statewide prevention work anecdotally observed a similar increasing morbidity and mortality pattern at their respective children’s hospitals.^2,3^ The Timely Recognition of Abusive Injuries (TRAIN) Collaborative was a quality improvement (QI) collaborative of 6 Ohio children’s hospitals created in 2015 to address this concern. The goals of the TRAIN Collaborative were to improve providers’ ability to recognize and respond to injuries concerning for physical abuse in young infants. The TRAIN Collaborative adopted the Institute for Healthcare Improvement’s (IHI) highly successful Breakthrough Series (BTS) Collaborative Model. This model provides a paradigm for partnership between interested organizations to facilitate iterative learning from each other and recognized experts in areas of desired improvement.^15,16^

**Fig. 1.**
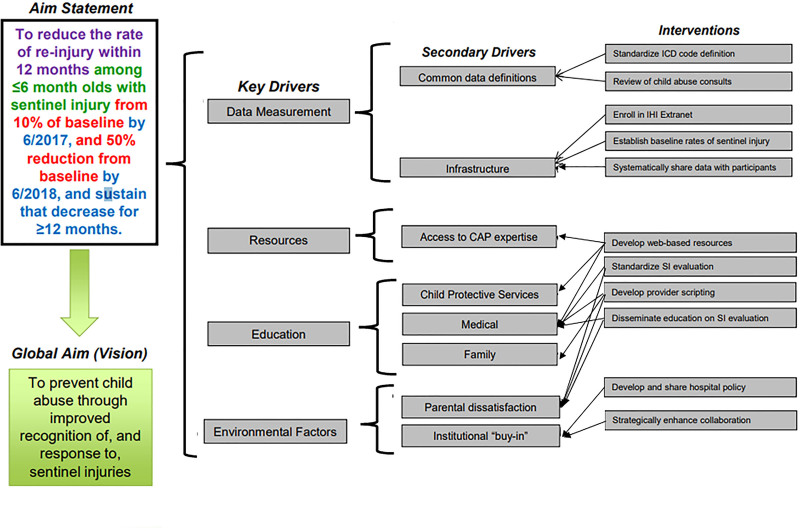
TRAIN global key driver diagram.

This report focuses on the primary phase of TRAIN, during which the collaborative’s global aim was reducing child abuse by improving recognition of and response to injuries concerning for physical abuse in infants 6 months and younger (Fig. 1). This study aimed to reduce the reinjury rate within 12 months among infants ≤6 months by 10% of baseline within 1 year, and 50% reduction from baseline within 2 years, and sustain that decrease for over a year.

## METHODS

Using the IHI’s BTS model, the TRAIN Collaborative engaged 6 children’s hospitals in Ohio, including child abuse pediatricians, pediatric emergency medicine physicians, social workers (SWs), nurses, and child-protective services (CPS) representatives. All hospitals are in urban settings, 5 are free-standing, and 1 is within an adult hospital; they vary in size from approximately 150 to over 600 beds. Each hospital has outpatient primary and subspecialty pediatric care, inpatient pediatric hospital teams, urgent care clinics, and at least 1 emergency department (ED). The local institutional review board of each hospital unanimously determined that this project was exempt from review.

Following the BTS model process, collaborative members developed standardized data definitions, including International Classification of Disease codes to identify injury in infants adapted from the work of Lindberg et al.^11^ (**See Appendix 1, Supplemental Digital Content,** http://links.lww.com/PQ9/A462.) The 6-month age cutoff was selected to exclude ambulatory infants who may sustain bruising or other superficial cutaneous injuries with increased mobility. For each patient identified, a physician or SW from that hospital verified the documented injury and coding accuracy via manual chart review. Patients were excluded if no injury was documented in the chart or if the injury was determined not to be concerning for abuse, including injuries that were birth-related, motor vehicle collision-related, caused by an animal, or sustained in the health care setting. This chart review occurred for the initial injury and any subsequent reinjury. Collaborative members from each hospital contributed deidentified data to a shared database hosted by IHI. These data established baseline and follow-up recurrent injury rates. Child abuse experts aided in developing an evidence-based standardized evaluation for infants identified to have injuries concerning for physical abuse; this evaluation included a thorough cutaneous and intraoral examination, skeletal survey, and psychosocial assessment.^10^

The IHI BTS model has collaborative members meet for learning sessions to exchange ideas and experiences, review data, and plan rapid improvement cycles for intervening action periods. Collaborative members used the initial learning session to identify opportunities specific to their institution to recognize injuries concerning for child physical abuse. Changes were made during subsequent action periods, and collaborative members reconvened multiple times to present their successes and obstacles to improvement. These sharing sessions helped members at other institutions make similar, beneficial changes. Every hospital came to the collaborative with a unique existing process. Therefore, different opportunities for intervention were identified and implemented at each institution (Table 1). The following are the most widely accepted interventions implemented at each hospital and adopted at other institutions due to their success.

**Table 1.** TRAIN Collaborative Interventions

Site	Intervention
All 6 institutions	Evidence-based standardized evaluation of infants with suspected NAT
	Educational updates for frontline clinical staff on signs of NAT in infants
Institution 1	Signage highlighting indications for skeletal survey
	Recurrent education during daily nursing huddles
Institution 2	Order sets for recommended laboratory work and imaging in infants with suspected NAT
	Development of scripting for providers and SWs to discuss concerns of NAT with caregivers
Institution 3	Assessed provider awareness of injuries concerning for NAT using a survey
	Development of education specifically around deficits identified in the survey
Institution 4	EHR-based reminder of required social work involvement for patients with concern for NAT
	Scripted questions for providers to standardize history-taking for patients with concern for NAT
Institution 5	Improved cutaneous examinations by encouraging undressing of all infants for examination
	Development of standardized EHR language to support documentation of examination
Institution 6	Educational efforts of all ED staff followed by follow-up survey to assess understanding

NAT, non-accidental Trauma.

Institution 1 developed signage highlighting the need for imaging, specifically skeletal surveys, for infants with injuries concerning for physical abuse. Signs were displayed in physician areas of EDs, urgent care units, and primary care centers. Staff were educated on injuries concerning for physical abuse in infants in monthly department meetings and daily nursing huddles.

Institution 2 created order sets that included recommended laboratory work and imaging to support completing the evaluation of these patients. Additionally, scripting was developed for medical providers and SW to use when discussing concerns and critical medical evaluation with caregivers.

Institution 3 utilized a survey to assess medical providers’ awareness of injuries concerning for nonaccidental trauma in infants and found that familiarity was low. They then implemented education around these concerning injuries and appropriate evaluation for occult injuries.

Institution 4 expanded SW involvement by requiring SW consultation for every infant with a concerning injury supported by a pop-up reminder in the electronic health record (EHR). Furthermore, ED physicians used scripted questions to improve the quality of injury history assessments done on infants seen with a concerning injury.

Institution 5 focused on improving cutaneous examinations in infants by ensuring all infants presenting for care have a thorough, undressed examination. They also developed standardized EHR verbiage to support documenting the complete cutaneous examination.

Finally, institution 6 provided section-wide education to ED staff on injuries of concern for abuse in infants and gauged understanding with a follow-up survey.

Each institution systematically collected data and calculated baseline and monthly metrics as described by Thackeray et al.^17^ All 6 hospitals collected the number of injuries identified in infants 6 months old and younger as the process measure. The outcome metrics were designed to look prospectively at reinjury frequency among infants. The outcome metrics were the rates of children presenting with an injury concerning for abuse within 3 and 12 months after a prior SI sustained between 0 and 6 months of age. Baseline data were collected from July 2013 to June 2014 to allow sufficient time to monitor reinjury rates as children aged. Postintervention data collection occurred from July 2015 to May 2018; these data were plotted in a c-chart to measure the number of children with injuries across the collaborative monthly. Reinjury rate was monitored in p-charts and demonstrated the total number of infants 0–6 months presenting with a second injury within 3 and 12 months. Of note, data were missing from 2 sites for 3-month reinjury data and a third site for 12-month reinjury data due to loss of staff support for data review, and those sites were excluded from these analyses.

Each site implemented statistical process control methods to monitor the impact of changes in process on outcome measures. Data from all hospitals were aggregated and analyzed in control charts. Upper and lower control limits were added to reflect anticipated variations in monthly data. Using Nelson’s rules, the collaborative assessed control charts for significant change and established a new centerline with substantial process shifts.^18^

## RESULTS

**Fig. 2.**
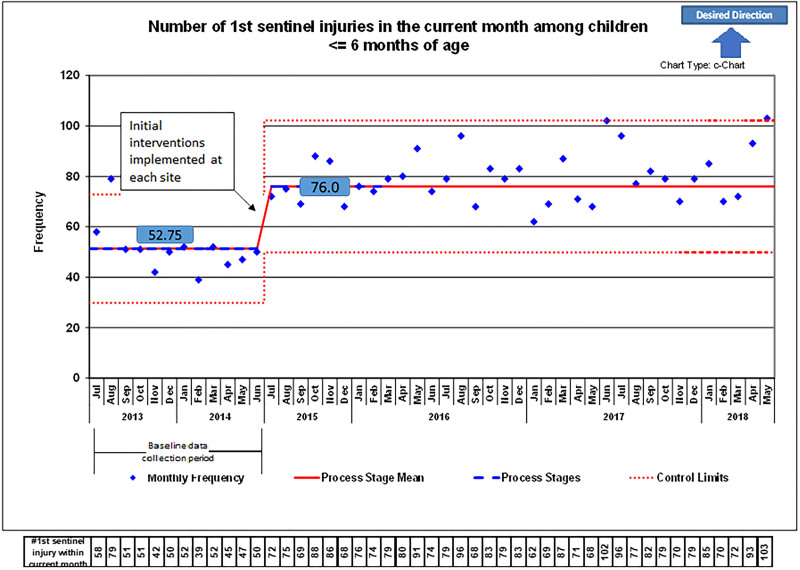
C-chart of the number of first concerning injuries in the current month among infants ≤6 months.

While each institution implemented different interventions, they started simultaneously in July 2015. Due to the low frequency of very young infants presenting with injuries at any individual hospital, data were aggregated from all institutions; this also helped identify baseline rates of injuries in young children across the state. Among all sites, the first process stage identified 616 infants (6 months and younger) with an initial injury during the preimplementation period from July 2013 to June 2014 (Fig. 2). This value is reflected as a baseline frequency of approximately 53 children identified with injuries per month. The initial intervention for all hospitals in July 2015 was educating on the importance of recognizing and responding to injuries in infants 6 months and younger. An upward centerline shift followed this intervention to a frequency of 76 children with injuries identified monthly, reflected in the second process stage. In total, 2785 infants with injuries were identified during the postimplementation period, which ended in May 2018.

**Fig. 3.**
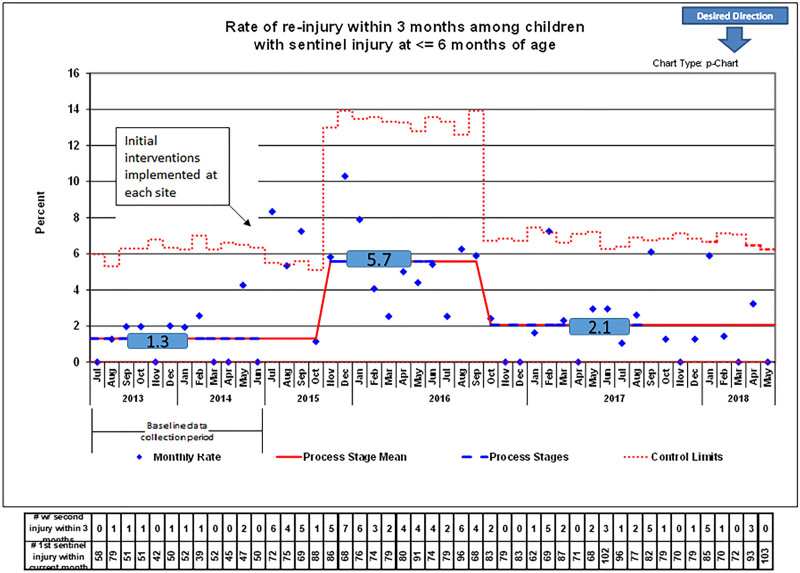
P-chart of reinjury rates within 3 months of infants with a concerning injury at ≤6 months.

The first outcome metric measured reinjury prospectively by following the reinjury rate in children within 3 months of being seen for an initial injury. For example, a child would be included in this measure if they had an injury at 4 months and then presented for a second injury at 6 months. Because of the need for time to elapse to monitor for reinjury in these children, the last month data available for this measure were May 2018. Like the process measure of identifying injuries, the p-chart (Fig. 3) shows an increase in the rate of children seen for reinjuries when the collaborative was initiated from 1.3% during the preimplementation period to 5.7% 4 months following implementation in the second-process stage, a change that was sustained for 11 months. The centerline subsequently shifted to a reinjury rate of 2.1% in October 2016, a change sustained through the study conclusion reflecting the impact of the interventions over time.

**Fig. 4.**
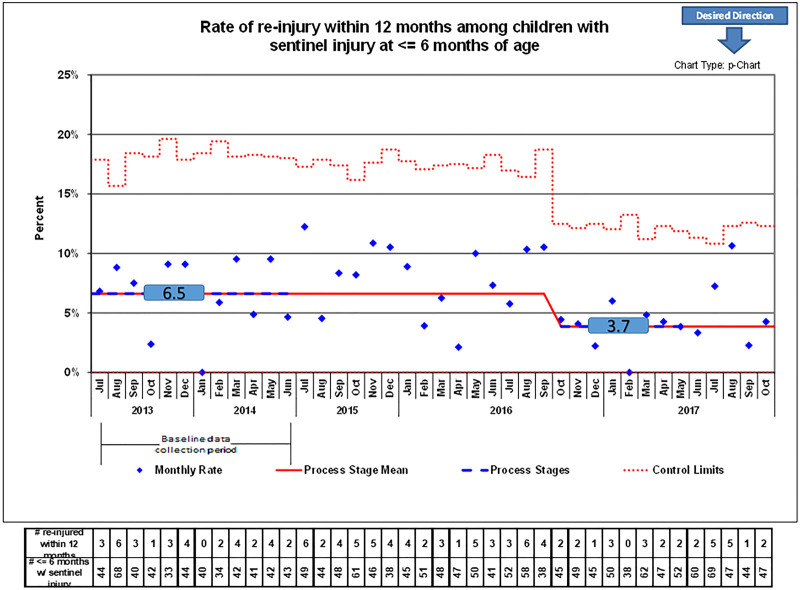
P-chart of reinjury rates within 12 months of infants with a concerning injury at ≤6 months.

The second outcome metric measured reinjuries prospectively for a more extended time by following the reinjury rate within 12 months of an initial injury. Again, due to the time lag needed to collect these data and retrospectively identify an initial injury, the last month available for this measure was October 2017. As with the first outcome metric, the p-chart (Fig. 4) displays a decrease in the rate of children seen for reinjuries once hospitals implemented TRAIN Collaborative interventions from 6.5% to 3.7% after 15 months. This change was sustained for the remainder of the study.

Data were available from all sites for the process measure. Two sites were missing complete data for the 3-month outcome measure. A third site was missing data for the 12-month outcome measure as data collection at that site ended prematurely. Data from these sites were not included in 3-month and 12-month outcome measures analyses. Of note, because of the rarity of the injuries, each site had months with no concerning injuries identified.

## DISCUSSION

TRAIN is the first multicenter QI collaborative established to improve recognition of and response to injuries in infants that may be indicators of child physical abuse. By engaging 6 children’s hospitals in this initiative, we developed several strategies unique to each institution to facilitate change and reduce recurrent physical abuse. After initiating interventions, we saw an increase in identified injuries across all 6 hospitals, likely related to providers being more aware of the concerning nature of these injuries. This trend remained constant for the entire study period and potentially reflected improved detection of child physical abuse, which has been an elusive goal. The reinjury rate then significantly declined after about 1 year. The impact of these interventions is further borne out in our second outcome metric, 12-month reinjury data, which also demonstrated a significant reduction (downward shift). These decreases in reinjury rates likely reflect improved identification of injuries leading to evaluation for additional injuries and engagement with partner community agencies that can facilitate improving children’s safety and preventing further harm.

QI methodology is an emerging tool to improve the care of children with potential physical abuse. Previous studies have described the use of QI resources at single institutions to improve the completion of skeletal surveys for infants with extremity fractures^19^ and the frequency of use of a child abuse screening tool.^20,21^ Moles et al^22^ engaged 16 providers nationally using a series of rapid cycle interventions to improve the quality of documentation of physical abuse assessments. We utilized IHI’s BTS model to engage 6 hospitals in improving identification and response to concerning injuries. This project is the first to apply this approach to child abuse identification and prevention. According to the model, each hospital developed unique interventions adapted to their specific institutional resources/needs and then shared successes and challenges with other hospitals to expedite improvement. For example, one intervention common to every hospital included specific education for ED providers about physical abuse in infants with minor injuries and appropriate evaluation of these children. Consistent with the literature that provider education is a barrier to identifying and evaluating these injuries, more injuries were identified once education about the significance of these injuries occurred.^9^ The learning sessions during the collaborative allowed for the exchange of ideas between hospitals to continue promoting the identification of injuries. For example, one hospital developed signage posted in clinician work areas reminding providers to order skeletal surveys for infants with injuries concerning for physical abuse. Other hospitals adopted this successful approach and enhanced it; one added hospital-specific data on the percentage of eligible infants receiving skeletal surveys. Another added graphics and a mnemonic to improve the internal marketing of the tool.

Other barriers to identifying injuries concerning for abuse noted in the literature include the time required to discuss these concerns with caregivers and provider knowledge of recommended evaluation for occult injuries.^9,23^ In this study, identifying concerning injuries improved with increased access to SW to enable discussions with families and implementing order sets in the EHR to facilitate completing appropriate laboratory and imaging workup.

The outcome of the TRAIN Collaborative demonstrates that with cooperative effort and relatively simple interventions (eg, targeted provider education, signage outlining evaluation of children with concerning injuries, and standardized documentation for the complete cutaneous examination), more injuries potentially concerning for physical abuse can be identified and recurrence of injuries in young infants can be reduced. The critical first step of improving injury identification initiates a cascade that reduces recurrence. The more injuries identified, the more often appropriate evaluations for injuries can be completed. Although not specifically studied in this collaborative, increasing identification of concerning injuries will facilitate appropriate reports for suspected physical abuse to local CPS agencies to help improve children’s safety. In addition, CPS engagement can facilitate increased safety of a child’s environment through various interventions, including parenting support, safety planning, and/or placement with alternative caregivers, either kinship or foster caregivers; these interventions can make it less likely that the child will sustain recurrent injury. It has been well demonstrated that young children who remain in an environment where they have been physically abused are more likely to suffer subsequent and potentially life-threatening abusive injuries.^2,3,12^ In this study, our interventions led to detecting more minor injuries concerning for abuse and reducing the recurrence of injury. Furthermore, these interventions served as a foundation for institution-wide QI work to reduce missed abuse at one of the participating hospitals.^24^

Engaging 6 hospitals in a QI effort was not without challenges, and this effort served as a learning experience in QI work. Understanding the roles of each team member in the project from every institution is critical, especially as there will be turnover across long-term projects that can significantly impact all aspects of the project, from the implementation of interventions to data collection and analysis. Fully clarifying existing practices at each site and the capacity for change of these practices also proved essential; this includes engaging stakeholders from every clinical area that may be involved with the recommended interventions. While having variation in intervention across sites introduces noise in comparing the success of each intervention, we believe that the encouraging outcomes demonstrated are at least partially the result of tailoring interventions to opportunities identified at each hospital.

Every institution entered the collaborative with varying practices for evaluating children with physical abuse concerns and had different access to child abuse pediatricians and SW support. Lacking a child abuse pediatrician and SW proved a significant barrier for 2 hospitals to identify suspected physical abuse and gather information necessary for this study. Thus, we had missing data from these institutions for reinjury outcome metrics. For 12-month reinjury data, a third hospital could not commit staffing to participate in data collection long enough to have meaningful data to contribute to this measure. Therefore, missing reinjury data were not included in 3-month and 12-month outcome measures to avoid underestimation of the number of children who were represented with concerning injuries; however, these 3 institutions had much lower patient volumes than remaining hospitals. Thus, the likely impact on outcomes is negligible.

While we saw a significant increase in the number of injuries identified, these data did not clarify how many additional injuries were missed. For example, many types of injuries we sought to identify are, by definition, minor and spontaneously resolved without medical intervention, making the opportunity for detection by a medical provider fleeting. Furthermore, some infants who suffered a recurrent injury could have sought care at a different institution and been missed in calculating 3-month and 12-month rates; data were deidentified in our database as per interinstitutional agreements precluding our ability to track patients across hospitals. Therefore, data may be incomplete and not entirely representative of all children with initial minor injuries concerning for physical abuse or subsequent reinjury.

Finally, only children’s hospitals participated in the TRAIN Collaborative. However, unpublished data from the Ohio Hospital Association (personal correspondence, 2014) indicate that approximately half of the infants in Ohio present to community hospitals and outpatient medical facilities without specialized pediatric providers and the capability to complete an appropriate workup for suspected physical abuse or SW support. Therefore, raising awareness in those settings would likely require a different approach.

Recognizing physical abuse in infants is critical to keeping children safe and preventing recurrent and escalating injuries. The TRAIN Collaborative demonstrated the IHI BTS model could be effectively deployed to detect injuries potentially concerning for child physical abuse and reduce subsequent injuries in this population. While hospitals in the collaborative initially utilized different interventions unique to needs identified at each institution, successful interventions were shared across the collaborative and adopted at other sites. Thus, the overall impact of the TRAIN project was a reduction in recurrent injuries. These findings highlight the need for ongoing education and support resources for medical providers on the significance of minor injuries in young infants. Furthermore, this study supports the critical role the hospital-based child protection teams, including child abuse pediatricians and SW, play in improving the detection and reducing the recurrence of abusive injuries. Future studies must examine how similar QI measures in other medical settings could help detect injuries in infants and reduce reinjury rates.

## ACKNOWLEDGMENTS

The authors thank the entire Timely Recognition of Abuse Injuries (TRAIN) Collaborative, including the principal TRAIN investigators as follows: Emily Eismann, MS, and Kathi Makoroff, MD, MEd (Cincinnati Children’s Hospital Medical Center, Cincinnati, OH); Christine Perebzak, MSN, RN, PCNSBC (Akron Children’s Hospital, Youngstown, OH); Lori Vavul-Roediger, MD (Dayton Children’s Hospital, Dayton, OH); Michelle Meehan, MSW, LSW, and Julie Vierling, MSW, LISW (ProMedica Toledo Children’s Hospital); and Grace Kim, MD (Rainbow Babies & Children’s Hospital, Cleveland, OH). The authors also thank Carrie Baker and Naomi Makni, MHA, for their assistance as project coordinators and the Ohio Children’s Hospital Association for its support. In addition, the Office of the Ohio Attorney General Mike DeWine funded the TRAIN Collaborative.

## DISCLOSURE

The authors have no financial interest to declare in relation to the content of this article.

## Supplementary Material


